# Distinct evolutionary patterns of *Neisseria meningitidis* serogroup B disease outbreaks at two universities in the USA

**DOI:** 10.1099/mgen.0.000155

**Published:** 2018-04-04

**Authors:** Li Hao, Matthew T. G. Holden, Xin Wang, Lubomira Andrew, Sabine Wellnitz, Fang Hu, Melissa Whaley, Scott Sammons, Kristen Knipe, Mike Frace, Lucy A. McNamara, Paul Liberator, Annaliesa S. Anderson

**Affiliations:** ^1^​ Vaccine Research & Development, Pfizer Inc, 401 N. Middletown Rd, Pearl River, NY 10965, USA; ^2^​ School of Medicine, University of St. Andrews, St. Andrews, UK; ^3^​ Division of Bacterial Diseases, Centers for Diseases Control and Prevention, Atlanta, Georgia, USA

**Keywords:** serogroup B *Neisseria meningitidis*, disease outbreak, multi-locus sequence typing (MLST), whole genome sequence (WGS), single nucleotide polymorphism (SNP), vaccine

## Abstract

*Neisseria meningitidis* serogroup B (MnB) was responsible for two independent meningococcal disease outbreaks at universities in the USA during 2013. The first at University A in New Jersey included nine confirmed cases reported between March 2013 and March 2014. The second outbreak occurred at University B in California, with four confirmed cases during November 2013. The public health response to these outbreaks included the approval and deployment of a serogroup B meningococcal vaccine that was not yet licensed in the USA. This study investigated the use of whole-genome sequencing(WGS) to examine the genetic profile of the disease-causing outbreak isolates at each university. Comparative WGS revealed differences in evolutionary patterns between the two disease outbreaks. The University A outbreak isolates were very closely related, with differences primarily attributed to single nucleotide polymorphisms/insertion-deletion (SNP/indel) events. In contrast, the University B outbreak isolates segregated into two phylogenetic clades, differing in large part due to recombination events covering extensive regions (>30 kb) of the genome including virulence factors. This high-resolution comparison of two meningococcal disease outbreaks further demonstrates the genetic complexity of meningococcal bacteria as related to evolution and disease virulence.

## Data Summary

Assembled contigs of isolates have been deposited in GenBank, BioProject ID: PRJNA395340 (http://www.ncbi.nlm.nih.gov/bioproject/395340).

**Outcome**

Meningococcal disease outbreaks in universities, the military and other organization-based settings present a significant public health concern. Genetic characterization of outbreak isolates can provide important insight into the origin and mechanism(s) of virulence of the bacteria, as well as providing information that can potentially impact infection control and vaccine design. Reported here is a case study using whole-genome sequencing (WGS) to analyse disease strains from two independent university outbreaks in the USA. Detailed comparison of WGS data identified distinct evolutionary patterns between the two groups of outbreak strains. Most notably, examples of homologous recombination events were evident among disease outbreak isolates from one university but not the second, where all outbreak isolates could be attributed to a single phylogenetic clade. This study also illustrates that a combination of both short- and long-read WGS platforms can add significant value to the genetic investigation of the epidemiology of disease outbreaks.

## Introduction

Meningococcal disease has a rapid onset and potentially devastating consequences, with high case-fatality rates as well as significant sequelae amongst survivors [1]. In developed countries, disease incidence is highest in infants and young children (<5 years of age), but peaks again among adolescents and young adults [2, 3]. Groups at increased risk include military recruits and students residing in close quarters such as dormitories [4]. *Neisseria meningitidis*, a leading cause of bacterial meningitis, is classified into 12 serogroups defined by the structure of the capsular polysaccharide [2]. Licensed capsular polysaccharide and capsular polysaccharide conjugate vaccines provide protection against four of the major invasive serogroups (A, C, Y and W) [2]. In contrast, the *N. meningitidis* serogroup B (MnB) polysaccharide is composed of polysialic acid repeating units that are similar to structures found on human neuronal cells (particularly during fetal development) and are not suitable as vaccine antigens [5]. Two licensed MnB vaccines [6, 7] are instead comprised of outer-membrane protein antigens.

Meningococcal disease outbreaks that occur at universities can pose significant challenges for infection control and health management due to social behaviour tendencies of young adults and the size and dynamics of the student population. In the USA, students entering college have been recommended to receive a quadrivalent meningococcal conjugate vaccine (MenACWY) since 2000. Since this time, the incidence of invasive infection from these serogroups has declined [8, 9]. However, vaccines providing protection against serogroup B were not commercially available in the USA until late 2014. Since 2008, MnB outbreaks have been reported in at least eight different universities across the USA [10]. The Centers for Disease Control and Prevention (CDC) has recently modified the definition of what constitutes an outbreak of *N. meningitidis* serogroup B disease to include ≥2 cases in an organization with a population of <5000 people (or 3 cases in a population ≥5000 people) in a period of 6 months or less (https://www.cdc.gov/meningococcal/outbreaks/).

During 2013, MnB disease outbreaks were reported at two universities in the USA (https://www.cdc.gov/vaccines/acip/meetings/downloads/min-archive/min-2014-10.pdf). Between March 2013 and March 2014, seven students at University A and two individuals with epidemiologic links to University A students were diagnosed with meningococcal disease and serogroup B strains were recovered from eight cases. Cases of disease were protracted over two academic years. Disease incidence at this university reached 134/100 000 population, nearly 1400 times greater than the disease incidence expected in the USA at the time [11]. Four cases of meningococcal serogroup B disease were identified at University B during November 2013. As a result of these two outbreaks, 4CMenB, a serogroup B vaccine that at the time was only licensed ex-USA, was deployed under an Investigational New Drug (IND) application to prevent further cases [11, 12]. In response to these two university outbreaks, the MnB vaccines Trumenba (bivalent rLP2086) and Bexsero (4CMenB) [13] were granted accelerated approval by the US Food and Drug Administration (FDA) for individuals ≥10 years of age in October 2014 and February 2015, respectively [6, 7].

*N. meningitidis* infections are considered as reportable diseases in many regions of the developed world. As part of routine microbiological surveillance for the purpose of monitoring disease prevalence and outbreak control, strains are also typed genetically. Historically, pulsed-field gel electrophoresis (PFGE) profiling of bacterial strains has been used widely for outbreak investigations [14, 15]. More recently, sequence-based genetic typing techniques such as multi-locus sequence typing (MLST) [16] and fine typing of individual virulence factors such as porin A (PorA) and porin B (PorB) have been used for global surveillance of this pathogen [17]. However, isolates collected from a disease outbreak are typically highly related and traditional molecular typing approaches are unable to provide sufficient resolution for detailed comparative analysis.

The rapid advancement of next-generation sequencing (NGS) technology and the development of sequence assembly algorithms have transformed the way molecular epidemiology is used to study populations of bacterial pathogens. Recent studies [18–20] illustrated the value of whole-genome sequencing (WGS) in rapid, accurate and comprehensive characterization of bacterial strains in clinical settings. WGS provides ultimate resolution and an opportunity to differentiate highly related strains and, therefore, enhance disease surveillance. Here, we present a retrospective analysis of meningococcal isolates from these two university outbreaks using WGS data to define the relatedness and evolutionary patterns within the respective subpopulations of outbreak disease isolates.

## Methods

### *N. meningitidis* outbreak and non-outbreak reference isolates

Meningococcal isolates included in this study were initially identified and characterized at state public health laboratories, after which isolates were sent to the CDC and confirmed by slide agglutination serogrouping and real-time PCR [21].

Eleven MnB isolates from eight of the individuals diagnosed with meningococcal disease at University A [March 2013 to March 2014, including independent isolates from the blood and cerebrospinal fluid (CSF) of 3 patients] were collected. Four MnB isolates were collected from students at University B, a single isolate from each of the four patients diagnosed with meningococcal disease during November 2013, together with an outbreak-associated isolate (PMB5018) collected in March 2013 from a fifth patient affiliated with the same university.

MnB isolate PMB5266 was selected as a reference for the University A outbreak. This isolate was originally collected from a patient diagnosed with meningococcal disease during 2013 in the USA and with no known epidemiologic link with University A. It was selected as it shares the same sequence type (ST), *porB* and *fetA* genotype as the University A outbreak isolates. MnB isolate M5178 was selected as the reference for the University B outbreak. Isolated in 1998 from a patient in Oregon with meningococcal disease, M5178 shares the same ST, *porB* and *fetA* genotype as the University B outbreak isolates.

### Molecular typing of outbreak isolates

Additional strain characterization, including PFGE, MLST and molecular typing of genes coding for factor H binding protein (fHBP), Neisseria adhesin A (NadA), Neisserial heparin binding antigen (NHBA) and porin A (PorA) was performed as described previously [15, 22, 23].

### Whole-genome sequencing

#### MiSeq, WGS

Genomic DNA from all outbreak and outbreak-associated MnB isolates was extracted and then sequenced on the Illumina MiSeq, with 2×250 bp paired-end sequencing chemistry as described previously [20].

#### PacBio WGS

Three of the outbreak isolates (a single representative from the University A outbreak and two from the University B outbreak) and PMB5266 (the University A reference strain) were selected to be sequenced with the Pacific Biosciences (PacBio) RSII instrument using P4-C2 sequencing chemistry. Genomic DNA was extracted using an ArchivePure DNA purification kit (5 PRIME) according to the manufacturer’s instructions. One SMRT Cell was run per isolate. The average read length was 6440.75 bp, and the average number of reads was 105 464 per SMRT Cell.

### Genomic analysis of outbreak strains

For MiSeq data, *de novo* assembly was performed using the CLC Genomic Workbench (v. 6.5.1) with default settings. Generally, the assembled MnB genomes contained 200–300 contigs, with a total size of ~2 Mbp per genome. The coverage depth ranged between 60× and 329× (Table S1, available in the online version of this article). The PacBio data was assembled using PacBio’s Hierarchical Genome Assembly Process v3 (HGAP). Three of the isolates were assembled into a single contig, while the fourth was assembled into four contigs. The average coverage was 128× (Table S2).

The BIGSdb (Bacterial Isolate Genome Sequence Database) [24] was implemented to extract and characterize genetic information corresponding to targets of interest, including genes in the capsular polysaccharide biosynthetic operon, housekeeping genes for MLST, *porA*, *porB*, *fetA* and genes coding for the protein antigens (fHBP, NadA and NHBA) of licensed MnB vaccines.

Reads from MiSeq data were mapped to the PacBio reference genomes using SMALT (http://www.sanger.ac.uk/resources/software/smalt/), and single-nucleotide polymorphisms (SNPs) were identified as described previously [25]. Insertions/deletions (indels) in the mapped data were detected using GATK [26]. Concatenated SNPs identified from genome-wide comparisons (excluding recombination regions) were used to generate a maximal-likelihood tree using RAxML [27]. Recombination events were identified using Gubbins (http://sanger-pathogens.github.io/gubbins/) [28]. Manual inspection of the mapped data in the BAM files using Artemis [29] was used to confirm the prediction of the SNPs and indel regions identified.

## Results

### Genetic analysis of University A outbreak isolates

Traditional molecular typing methods (PFGE and gene-specific sequence typing) were initially performed for each of the University A outbreak isolates. All 11 isolates collected from the eight patients shared the same profile: PFGE pattern 429, ST/clonal complex (CC) ST-409: CC41/44/Lineage3, PorA variant type P1.5-1,2-2, PorB variant type 3-82 and FetA variant type F1-5 (Table 1), illustrating the considerable genetic similarity among these outbreak isolates. As described previously [11], antigens associated with serogroup B vaccines were also identical among the 11 isolates: fHBP variant B153 (pubmlst variant: 276), NHBA peptide variant 2, PorA P1.5-1,2-2 and an absence of the gene coding for NadA.

**Table 1.** Molecular typing results for the University A outbreak isolates*

Patient ID	Strain ID	Source of specimen
P1	PMB5024	CSF
P2	PMB5023	CSF
P3	PMB5021	Blood
P3	PMB5022	CSF
P4	PMB5025	CSF
P5	PMB5026	Blood
P6	PMB5027	CSF
P6	PMB5028	Blood
P7	PMB5029	Blood
P7	PMB5030	CSF
P8	PMB5301	Blood

*All 11 isolates had the following profile: PFGE pattern, 429; ST/CC, ST-409: CC41/44/Lineage3; PorA variant type (VR1,VR2), P1.5-1,2-2; PorB variant type, 3-82; FetA variant type, F1-5; fHBP variant, B153; NHBA peptide, variant 2; NadA variant, null.

**Fig. 1.**
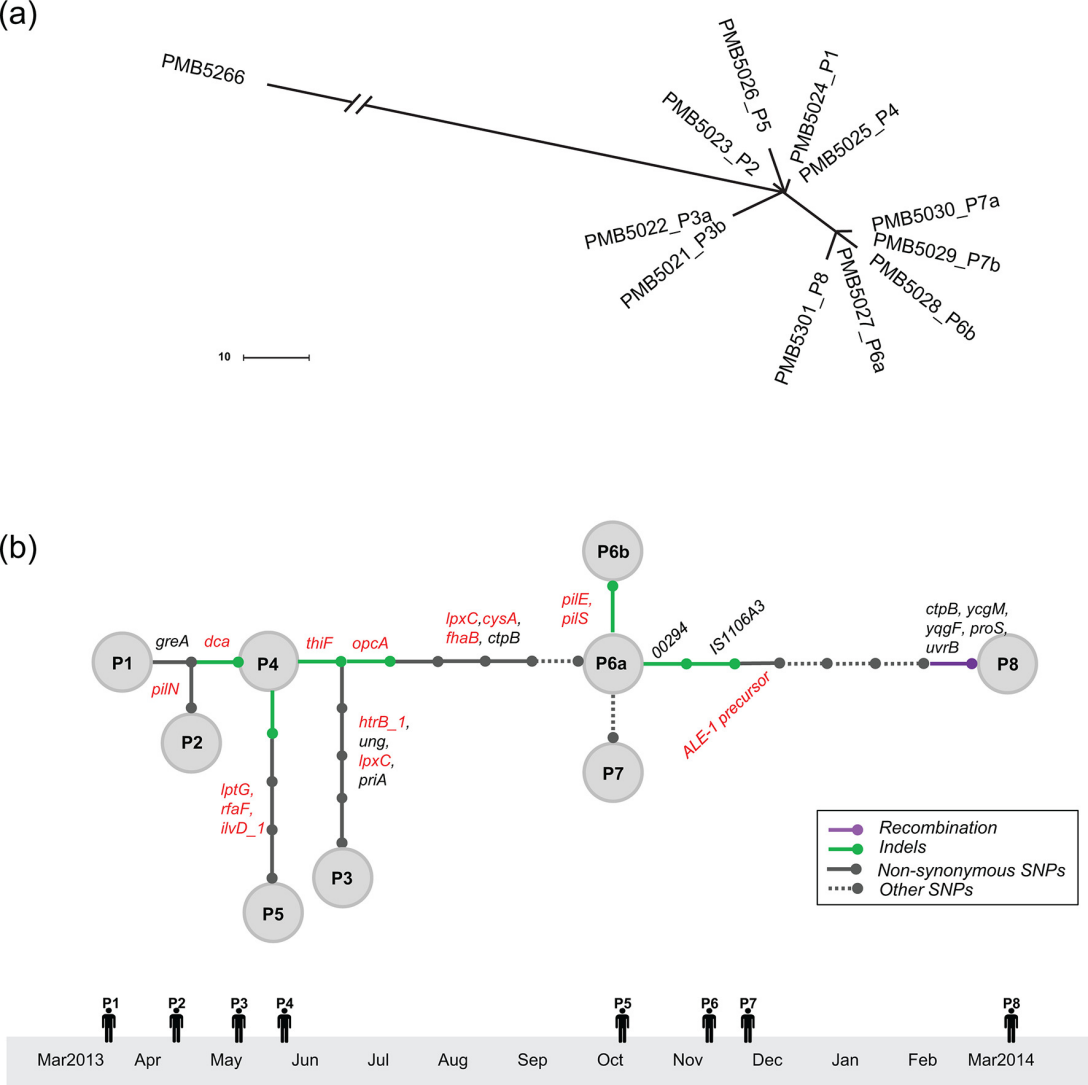
Whole-genome sequence based phylogeny and genetic transmission map among outbreak isolates from University A. (a) Maximum-likelihood phylogenetic tree generated using concatenated SNPs derived from genome-wide comparison (recombination events excluded). Scale bar relates branch length to the number of genetic changes. Isolate PMB5266 represents the MnB disease isolate that does not belong to the outbreak epidemic. For convenience of visualization, the long branch between PMB5266 and University A outbreak isolates was collapsed. (b) Transmission map was constructed using genetic and epidemiology data from the outbreak isolates, as detailed in Methods. Each node corresponds to an isolate(s) from an individual patient. Each bar represents a genetic change, and the types of genetic change (recombination events, indels, non-synonymous SNPs, other SNPs) are differentiated by line colour/style. Other SNPs, labelled as dashed lines, include synonymous SNPs and SNPs located in intergenic or promoter regions. Genes with changes which alter protein sequence (i.e. non-synonymous changes) are annotated alongside the bar. Red text is used to highlight known virulence genes.

The MnB invasive disease isolate, PMB5266, which was not part of the University A outbreak, was selected as a reference strain as it shared several genetic markers with the outbreak isolates, including MLST/CC, PorB, NHBA and NadA variant types. Comparative whole-genome SNP-based phylogenetic analysis (Fig. 1a) indicated that all 11 University A outbreak isolates were grouped into a tight cluster, well separated from PMB5266. In contrast to the numerous genetic changes identified between the outbreak isolates and the reference strain, detailed genome comparisons uncovered a total of just 18 SNPs (13 non-synonymous, 2 synonymous, 2 intergenic, 1 promoter region), seven indel events and one recombination event that distinguished the group of 11 outbreak isolates among themselves. The large contigs generated from the PacBio assembly of the PMB5026 outbreak isolate allowed for detailed mapping of individual genetic changes among the outbreak isolates. A genetic transmission map was reconstructed after integrating genetic and epidemiology data (Fig. 1b). Non-synonymous changes associated with genes involved in amino acid and lipid biosynthesis, such as *ilvD_1* (dihydroxy-acid dehydratase), *lpxC* (UDP-3-*O*-acyl-acetylglucosamine deacetylase), *htrB_1* (lipidA biosynthesis lauroyl acyltransferase) and *lptG* (lipopolysaccharide export system permease protein), were identified among the 11 outbreak isolates. Two strains (one each from blood and CSF) were isolated from each of three University A patients (P3, P6 and P7). High-resolution analysis of each pair of isolates identified only one deletion event for the pair of isolates collected from P6. The deletion occurred in the *pilE* and *pilS* genetic loci, a region known to be highly variable in *N. meningitidis*. No differences were noted when comparing the blood and CSF isolates from patients P3 or P7.

The CC41/44/Lineage3 is commonly associated with invasive disease, but ST-409 is relatively rare. We, therefore, sought to evaluate how these MnB outbreak isolates differed from another ST-409 isolate (PMB5266) that had caused sporadic disease. Using the PacBio long-read sequencing platform, WGS data was also generated for the non-outbreak reference isolate PMB5266. From pairwise alignments between PMB5026 (outbreak isolate) and PMB5266 (non-outbreak isolate), 99.8 % of the genomes are highly conserved, with an average of 99.2 % sequence identity across 38 conserved segments defined as locally collinear blocks (LCBs) that lack genome rearrangements. Detailed analysis uncovered a total of 17 recombination and 46 SNP/indel events which differentiate the PMB5026 outbreak isolate from the reference PMB5266. Several of these differences are positioned in genes coding for meningococcal virulence factors (e.g. fHBP, PorA, capsular polysaccharide) (Fig. S1a, Tables S3 and S4).

### Genetic analysis of University B outbreak isolates

Four MnB isolates were collected from students at University B, a single isolate from each of the four patients diagnosed with meningococcal disease during November 2013, together with an outbreak-associated isolate (PMB5018) collected 8 months earlier during 2013 from a fifth patient affiliated with the same university. Unlike the similar profile identified among the University A outbreak isolates, the four outbreak isolates from University B could be divided into two groups based on different PFGE profiles (467, 468) and PorB variant types (3–24, 3–461; Table 2). The outbreak-associated isolate PMB5018 shared the same profile as one of the two groups (PFGE-467 and PorB 3–24). All University B isolates were typed as ST-32 and CC32/ET5 complex, and contained genes that code for fHBP variant B24, NadA variant 1, NHBA variant 5 and PorA type P1.7,16-20 (Table 2).

**Table 2.** Molecular typing results of University B outbreak and outbreak-associated isolates*

Patient ID	Strain ID	Source of Specimen	PorB	PFGE
P1†	PMB5018	Blood	3–24	467
P2	PMB4478	CSF	3–24	467
P3	PMB4477	Blood	3–24	467
P4	PMB4479	Blood	3–461	468
P5	PMB5019	Blood	3–461	468

*All isolates had the following profile: ST, ST-32; CC, CC32/ET5 complex; fHBP variant, B24; NadA variant, 1; NHBA peptide, variant 5; PorA variant type (VR1,VR2), P1.7,16-20; FetA variant type, F3-3.

†Outbreak-associated isolate.

**Fig. 2.**
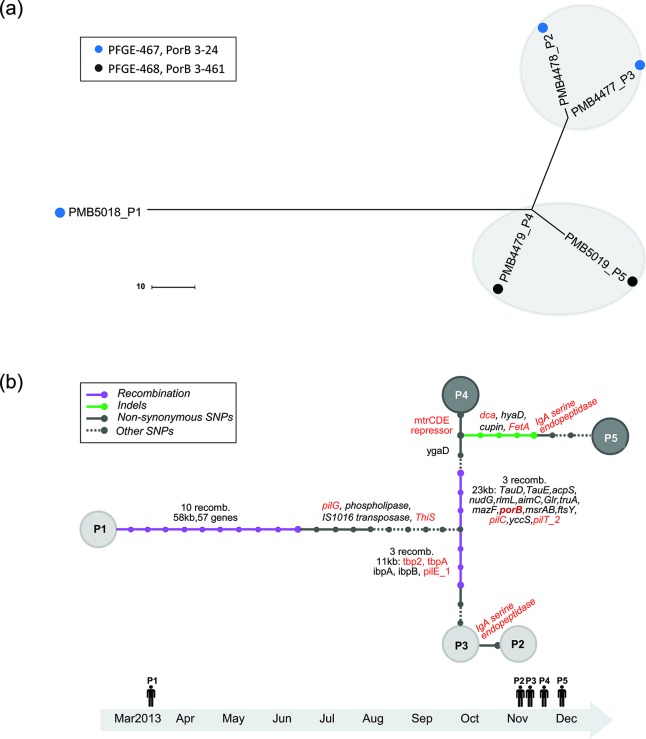
Whole-genome sequence based phylogeny and genetic transmission map among outbreak isolates from University B. (a) Maximum-likelihood phylogenetic tree generated using concatenated SNPs derived from genome-wide comparison (genetic changes associated with recombination events excluded). Scale bar relates branch length to the number of genetic changes. PMB5018 represents the outbreak-associated isolate, cultured from a patient nearly 8 months before the first outbreak isolate. (b) The transmission map was reconstructed using genetic and epidemiology data from the outbreak (or associated) isolates, as detailed in Methods. Each node corresponds to an isolate from an individual patient. Each bar represents a genetic change, and the types of genetic change (recombination events, indels, non-synonymous SNPs, other SNPs) are differentiated by line colour/style. Other SNPs, labelled as dashed lines, include synonymous SNPs and SNPs located in intergenic or promoter regions. Genes with changes which alter protein sequence (i.e. non-synonymous changes) are annotated alongside the bar. Red text is used to highlight known virulence genes.

WGS data for the four University B outbreak isolates and the outbreak-associated isolate were generated using the Illumina MiSeq short-read platform. The phylogenetic tree using SNPs derived from WGS comparisons among the four outbreak isolates (Fig. 2a) revealed two phylogenetic clades, consistent with the PFGE and PorB typing results. The genetic diversity within each of the two clades was small and comparable with the genetic diversity observed among the 11 isolates from the University A outbreak. The phylogenetic distance between the outbreak-associated isolate PMB5018 and the outbreak isolates was greater than the distance between the two outbreak clades (Fig. 2a). To accurately describe the genetic evolution of the two University B outbreak clades, a representative isolate from each was sequenced using the PacBio long-read sequencing platform (PMB4478 and PMB4479; Table S2). Unlike the University A outbreak, the evolution of the University B outbreak isolates was driven primarily by large recombination events (spanning close to 100 kb and including nearly 80 genes) (Fig. 2b). The two phylogenetic clades were the product of six recombination events that span genomic regions totalling >30 kb. The gene coding for PorB is located within one of the recombination blocks, consistent with the differential *porB* genotype noted earlier. In addition to these recombination events, 18 SNPs were identified among the University B isolates, nine of which resulted in non-synonymous changes.

Collectively, isolates from the University B outbreak are genetically related to isolates associated with prolonged increased MnB disease incidence in Oregon (referred to as the Oregon clone), sharing the same ST/CC and FetA and fHBP variant types [30, 31]. A representative Oregon clone (MnB strain M5178) was selected as a reference strain for high-resolution whole-genome comparative analysis with the University B outbreak isolate PMB4478. Syntenic regions between the two disease-causing isolates were identified covering approximately 95 % of the respective genomes, with 99.8 % pairwise nucleotide sequence identity. Deep analysis uncovered a total of 20 recombination events and 85 SNPs/indels that impacted >150 genes. Many of these genes are known to code for proteins associated with meningococcal virulence, including *capD*, *porA*, *tbp2*, *pilE* and *igA-*specific serine endopeptidase [32] (Fig. S1b, Tables S5 and S6).

## Discussion

Genetic characterization of disease-causing bacteria is fundamental for an understanding of pathogen evolution and the epidemiology of disease. MLST and antigen typing methods have been successfully exploited to characterize many large collections of invasive meningococcal disease (IMD) strains to probe molecular diversity from different geographical regions [33, 34]. However, strains associated with meningococcal disease outbreaks are typically highly related [35] (i.e. the same sequence type and clonal complex, ST/CC) and the detail afforded by these gene-based sequence typing methods is insufficient for meaningful comparative evaluation. WGS, powered by the rapid development of high-throughput sequencing technology platforms and *de novo* assembly algorithms, has been used more recently to characterize closely related outbreak isolates and study disease transmission [36–38]. While several serogroup B disease outbreaks have been documented in recent years in the USA and Europe [10, 11, 39, 40], comparative analysis of different outbreaks using WGS has not been reported previously. In this study, we used WGS to investigate the genetic diversity of MnB isolates from independent meningococcal disease outbreaks that occurred at two universities in the USA during 2013. While the outbreaks occurred during the same calendar year among individuals of the same age and with similar social tendencies, comparative genomic analysis indicated that the evolutionary patterns of the two collections of outbreak isolates were considerably different from one another. Even though the outbreak at University A was more protracted than the outbreak at University B, isolates from the University A outbreak were considerably more homogeneous with fewer SNP/indel changes. Conversely, the University B outbreak isolates could be divided into two distinct subpopulations distinguished from one another by large recombination events.

All isolates from the University A disease outbreak were typed as ST-409, a single locus variant of ST-44 and part of the ST-41/44/Lineage3 CC, one of the most prevalent and hypervirulent lineages globally since the 1970s [9, 41]. Although MnB disease outbreak strains typed as CC41/44 have been reported, none have belonged to ST-409. Among nearly 19 000 invasive meningococcal disease isolates catalogued at PubMLST.org, only 28 are typed as ST-409. Each of these is a serogroup B strain collected from patients in Europe or northern Africa. Genetically distinct, the University A outbreak strains and the ST-409 isolates at PubMLST.org express different fHBP (B153 vs subfamily A variants) and PorA variants. One of the University A outbreak strains (PMB5026) was sequenced using the PacBio platform and used as a reference for detailed comparative genomic analysis among all 11 outbreak isolates. A total of just 18 SNPs, seven indels and one recombination event were identified, an illustration of the clonal nature of the University A outbreak isolates. Transmission of *N. meningitidis* is via contact with droplets from the upper respiratory tract, typically resulting in colonization and asymptomatic carriage in otherwise healthy individuals. Under certain instances that are not well understood, *N. meningitidis* is capable of invading the human host, which then can manifest as life-threatening invasive disease. With no documented evidence of direct contact among the University A students diagnosed with meningococcal infections, the protracted course of the outbreak was consistent with multiple independent clonal transmission events from asymptomatic carriers in the population. In a recent study of a meningococcal disease outbreak among members of a large extended indigenous ethnic minority Traveller family [38], genotypes consistent with the disease-causing strains were identified as being carried asymptomatically in the population. Unfortunately, a study to examine carriage isolates among students at University A was not conducted.

The genome of *N. meningitidis* is highly dynamic, subject to homologous recombination and horizontal gene transfer events. Evidence of this was reported in the Traveller family study [38], where genetic diversity was noted among disease isolates, including potential evidence for inter-species horizontal gene transfer. This is in marked contrast to the clonal nature of the 11 disease isolates from the University A outbreak and the genetic stability of this outbreak cluster over two academic years. Within the two central STs of the ST41/44 clonal complex, ST-41 is largely associated with invasive disease, whereas ST-44 is more often associated with meningococcal carriage [42]. The ST-409 genotype of the University A outbreak strains is a single locus variant of ST-44 at *pdhC*, differing by just seven nucleotide substitutions each resulting in a synonymous change. Despite the prolonged epidemiologic persistence of ST-44 among asymptomatic *N. meningitidis* carriage isolates, disease outbreaks associated with ST-44 (or related STs, such as ST-409) have been rare.

Each of the University B outbreak and outbreak-associated isolates belongs to the ST-32/ET-5 clonal complex, a clonal complex associated with meningococcal disease since the 1970s, including a persistent epidemic in Norway that spanned >20 years [43] as well as prolonged MnB disease incidence in Oregon [30]. In contrast to the genetic homogeneity among University A outbreak isolates, isolates in the University B disease outbreak could be grouped into two phylogenetic clades, initially differentiated by their PFGE profile (types 467 and 468) and PorB variant type (3-24 and 3-461). Deeper analysis of WGS data from University B isolates uncovered six recombination events encompassing >30 kb (>1 % of genome) that differentiated the two clades from one another. Two of these recombination events each span regions >10 kb, a length which is much greater than the average recombination length for *N. meningitidis*, previously estimated to be 1.1 kb [44]. The four University B disease cases were diagnosed within a month of one another, and isolates representative of the two clades (PMB4477 and PMB4479) were cultured from patients with disease onset <1 week apart. There was no direct contact among any of the University B cases. Given this abbreviated timeline, it is likely that meningococci from both clades were circulating simultaneously among asymptomatic carriers in the population. Unfortunately, contemporaneous carriage isolates from asymptomatic University B students were not collected to provide additional insight.

The evolutionary dynamics of an outbreak can be shaped by several factors, including mutation rate, mode of transmission, adaptation to host environment and stochastic events. Owing to the extreme complexity of within-host evolution, it is challenging to identify the factor(s) primarily contributing to the distinct evolutionary patterns of these two University outbreaks. Main limitations of this study are that the sample size of each outbreak was small and asymptomatic carriage samples were not collected to permit a comparison with invasive cases.

Generally, meningococci associated with epidemics and outbreaks belong to more uniform clonal groups, compared with meningococci causing sporadic disease [45]. However, differences between sporadic disease and outbreak isolates from the same ST/CC group are poorly understood. Here, genetic differences (SNPs/indels) distinguishing the outbreak isolates from the two universities and the respective reference disease isolates were compiled (Tables S3–S6). Where available, the presumptive functions of the proteins identified in this comparative analysis were grouped into four categories: (i) surface/capsule/virulence factors, (ii) metabolism/pathway, (iii) transcription/translation/signalling and (iv) transport/other (Table S7). Included on this list are known virulence factors that mediate attachment of *N. meningitidis* to mucosal cells of the nasopharynx (e.g. pilin and associated surface proteins) and, therefore, colonization [32]. Similarly, antibiotic resistance resulting from the modification or regulation of efflux pump components can promote bacterial survival in the presence of therapeutic pressure [46]. The distribution of genetic events that differentiate the two collections of university outbreak isolates from the respective reference isolates among the four categories was similar for both disease outbreaks (Table S7). Specifically, genes related to intra- and extra-cellular signalling (including transcription and translation machinery) were most often identified as being different in the comparison between outbreak and the respective non-outbreak isolates. For each outbreak, only 9–12 % of the proteins impacted were related to modifications of surface and capsule proteins or virulence-associated factors, six of which were common to both outbreaks. These shared virulence factors include *porA, mviN, icsA, pilC, ctrD* and *ostA*, with most of the changes associated with the postulated genetic recombination events. Any link between these genetic differences and disease transmission remains to be demonstrated experimentally.

In contrast to the low discriminating power afforded by conventional typing methods (PFGE and MLST), WGS can provide the genetic fingerprint of an isolate. In this study, we took advantage of a hybrid WGS approach, using two sequencing platforms (PacBio and Illumina). Relatively inexpensive, Illumina sequencing was used to rapidly assess the relatedness of disease isolates. PacBio WGS of a representative isolate was used to generate a reference sequence, against which specific changes among related strains were identified and potentially associated with phenotype. There was high concordance across WGS data for the majority of isolates that were sequenced using both platforms (Table S8). Interestingly, ~5 % (120 kb) of the PMB5019 PacBio genome was not well covered by the Illumna MiSeq reads, an observation that highlights the value of obtaining reference genomes from long-read sequencing technology for this type of detailed analysis.

In response to the meningococcal disease outbreaks at both University A and B, two MnB vaccines, Trumenba (bivalent rLP2086) and Bexsero (4CMenB), were granted accelerated approval by the FDA in October 2014 and February 2015, respectively [6, 7]. The outer-membrane protein fHBP is an antigen included in both vaccines. Detailed epidemiological studies have illustrated that the gene coding for fHBP is present in the majority of MnB isolates [33, 47]. Expression and accessibility of the vaccine antigen on the bacterial pathogen to the host immune system is critical for vaccine-elicited antibodies to kill disease-causing meningococci [48]. Robust fHBP surface expression was detected on outbreak isolates from both University A and University B [49]. In exploratory serum bactericidal assays using human complement (hSBAs), sera from individual subjects vaccinated with Trumenba were able to kill representative outbreak strains from University A and B (hSBA response rates of 44.4–77.8 and 77.8–100 % following 2 and 3 doses, respectively) [49]. These data illustrate the functional activity elicited by Trumenba against strains expressing fHBP variants heterologous to the vaccine antigens (variant B153 from University A and variant B24 from University B). While there is no hSBA data available with Bexsero immune sera and the outbreak isolate from University B, a recent analysis revealed that 66.1 % of students vaccinated at University A were seropositive in hSBA against a representative University A outbreak strain after receiving two doses of the vaccine [50].

To our knowledge, this is the first comparison of the evolution of isolates from two independent MnB disease outbreaks. Disease isolates from the University A outbreak were clonal in nature. Illustrating the differential evolutionary patterns between these two outbreaks, the isolates collected in the University B outbreak could be divided to two phylogenetic clades, the product of several independent recombination events. We identified genetic changes associated with virulence factors and outer-membrane proteins. This study also illustrated the value of a hybrid WGS approach that combines long- and short-read sequencing technologies in the genetic investigation of disease outbreaks.

## Supplementary Data

808 Supplementary File 2
