# Supplementary material for: Distinct evolutionary patterns of Neisseria meningitidis serogroup B disease outbreaks at two universities in the USA
Source: Microb Genom. 2018 Apr 4;4(4):e000155. doi: 10.1099/mgen.0.000155 (PMC5989579; doi:10.1099/mgen.0.000155)
Supplement: Supplementary File 2 [file mgen-4-155-s001.pdf]

Supplement Fig. 1. Circular genome comparison of an outbreak isolate and the corresponding non-outbreak reference isolate from University A (a) and University B (b). The plot was generated using BRIG [51], which runs BLASTN to search the outbreak isolate genome against a reference genome. The concentric rings from innermost to outermost represent the following: Ring 1- Genome size and position; Ring 2 – GC content (peaks or valleys illustrate GC and AT rich regions respectively that differ from 50% GC content); Ring 3 – Position of predicted recombination events; Ring 4 – BLASTN results, with color saturation corresponding to the nucleotide sequence identity; Ring 5 – Position of SNPs/indels that either introduce amino acid substitutions or that map to within 100nt of the amino terminal end of open reading frames. The position of three known virulence factors (fHBP, PorA, and CPS) where recombination events differentiate the University A outbreak strain from the reference strain are highlighted in red font.

Supplemental Fig 1

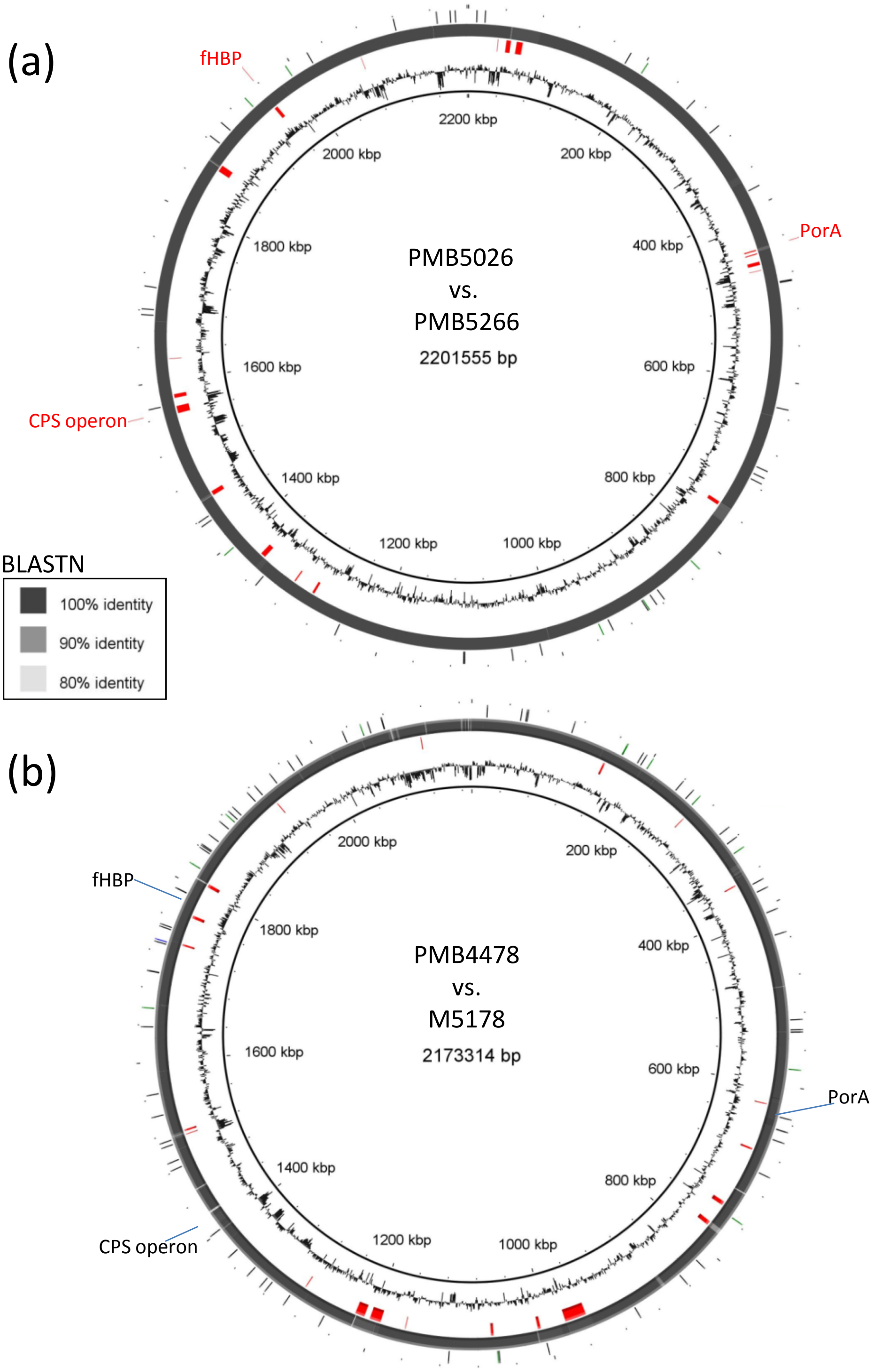

Supplemental Table 1. Assembly statistics of MnB outbreak strain s sequenced with Illumina MiSeq

MiSeq, PE, 2X250bp	Number of reads (PF)	Number of reads after merge	Mean read length (bp)	Number of assembled contigs	N50 (bp)	Average length of contig (bp)	Total length (bp)	Average Coverage
**University A**								
PMB5024	1,889,654	1,168,549	220	339	14,275	6,284	2,130,436	162
PMB5023	2,144,746	1,323,796	217	256	27,999	8,292	2,122,824	179
PMB5021	678,700	413,911	228	369	11,130	5,690	2,099,687	60
PMB5022	1,481,810	896,165	220	369	15,337	5,735	2,116,149	126
PMB5025	4,101,696	2,402,622	206	346	17,817	6,188	2,140,935	329
PMB5026	3,462,180	2,062,665	217	297	19,854	7,174	2,130,705	293
PMB5027	3,745,106	2,204,026	208	333	18,474	6,433	2,142,178	304
PMB5028	3,902,842	2,313,783	203	299	18,669	7,096	2,121,836	307
PMB5029	2,981,928	1,786,353	225	314	16,112	6,789	2,131,636	261
PMB5030	2,691,812	1,668,100	241	280	19,143	7,611	2,130,990	252
PMB5301	2,067,108	1,243,467	341	206	33,450	10,352	2,132,517	229
**University B**								
PMB5018	2,333,630	1,546,299	265	218	33,683	9,867	2,151,039	237
PMB4478	3,121,604	2,012,533	225	458	16,924	4,861	2,226,544	271
PMB4477	2,128,878	1,340,877	227	662	12,965	3,417	2,262,027	186
PMB4479	2,237,868	1,369,191	222	313	14,807	6,940	2,172,329	192
PMB5019	3,046,576	1,878,245	239	372	17,131	5,932	2,206,828	283

Supplemental Table 2. Comparison of WGS assembly statistics between Illumina MiSeq and PacBio platforms

	MiSeq (Illumina) - Short Read (2X250bp)					PacBio RSII (Pacific BioScience) - Long Read				
Sample Name	Number of assembled Contigs	Mean Coverage	Max Contig Length	N50 Contig Length	Sum of Contig Lengths	Number of Assembled Contigs	Mean Coverage	Max Contig Length	N50 Contig Length	Sum of Contig Lengths
PMB5026	297	293	61,491	19,854	2,130,705	4	70.81	803,368	767,488	2,228,024
PMB4478	458	271	52,626	16,924	2,226,544	1	195.63	2,246,541	2,246,541	2,246,541
PMB4479	313	192	57,276	14,807	2,172,329	1	144.62	2,243,872	2,243,872	2,243,872
PMB5266	251	101	100,816	36,483	2,112,265	1	99.82	2,206,764	2,206,764	2,207,764

Supplemental Table 3. Genetic changes identified from comparative WGS analysis of reference strain PMB5266 and University A outbreak isolate PMB5026. The list is restricted to SNP/indel events that alter protein sequences or are within 100 nucleotides of protein coding regions and have potential to alter gene expression.

Gene ID	Gene Name	Genetic Changes	Gene Description
00007	*pglE*	124 ins. CAACAAA	Glycosyltransferase
00018	*fbpB*	I443V	ABC transporter permease
00198	-	L36V	putative RNA-binding protein, YhBY family
00218	*nmgII*	promoter, C->T	DNA (cytosine-5-)-methyltransferase
00280	*pilK*	N144K	type IV pilus biogenesis protein
00331	-	V62I	hypothetical protein
00408	-	W20[STOP]	putative fimbrial assembly protein
00498	*ilvD_1*	M83I	phosphogluconate dehydratase
00499	*zwf*	V105A	glucose-6-phosphate dehydrogenase
00616	-	T118A	Perplasmic protein
00660	*hyaD*	102 del. CCC	glycosyl transferase family protein
00680	*lolE*	V211A	lipoprotein releasing system transmembrane protein
00687	*hom*	A322V	homoserine dehydrogenase
00695	*hsdM*	S85G	type I restriction-modification system methyltransferase
00817	*opcA*	promoter, del. CC	outermembrane protein
00875	-	68 del. AGCAAGCA	hypothetical protein
00886	*ackA_1*	R191H	acetate kinase
00895	*rfaF*	R81C	ADP-heptose--LPS heptosyltransferase II
00930	*lptG*	E35K	putative permease,Lipopolysaccharide export system permease protein
00940	-	promoter, C->T	hypothetical protein
01004	-	L292P	integral membrane protein,Predicted GTPase
01034	*hpuB_2*	386 del. C	hemoglobin receptor,Heme-repressible hemoglobin-binding protein
01080	*ttgA*	21 ins. GC	antibiotic resistance efflux pump component
01082	*acrR*	112 ins. T	mtrCDE transcriptional repressor,Potential acrAB operon repressor
01204	-	V142M	integral membrane transport protein,sucrose/H symporter
01306	*purB*	A48V	adenylosuccinate lyase
01350	*nqo3*	promoter, G -> A	NADH dehydrogenase subunit G
01377	*cotSA*	249 ins. GGG	pilin glycosyl transferase A
01383	*gyrB*	S609A	DNA gyrase subunit B
01391	*rnfB*	N146S	ferredoxin,Nitrogen fixation protein
01530	*siaC_1*	246 ins. A	polysialic acid capsule biosynthesis protein
01621	-	H157R	outer membrane protein/assembly factor
01627	*yfaY*	G162E	competence damage-inducible protein A
01661	-	L48I	hypothetical protein
01803	*ssa1*	94 del. GGG	extracellular serine protease precursor
01824	*slt*	V558A	transglycosylase,Soluble lytic murein transglycosylase precursor
01844	*lgtC_1*	54 ins. GG	lipooligosaccharyl-alpha-1
01884	*yggG*	promoter, del. TCC	peptidase, M48 family,Uncharacterized metalloprotease
01907	*dxs*	A531V	1-deoxy-D-xylulose-5-phosphate synthase
01926	*pilC*	27 del. CCC	type IV pilus assembly protein, pilC
01936	*OatA_2*	promoter, del. G	putative LipO-oligosaccharide acyltransferase
01957	-	Q322[STOP]	glycoside hydrolase family protein, pglH
02046	*dsbC*	A57T	thiol:disulfide interchange protein
02097	-	L167F	integral membrane protein
02109	-	V80A	integral membrane protein,Lipid A core - O-antigen ligase and related enzymes
02118	*hdaH*	C315Y	Histone deacetylase family protein

Supplemental Table 4. Recombination events identified from comparative WGS analysis of reference strain PMB5266 and University A outbreak isolate PMB5026. Only recombination blocks >100bp are listed.

Recombination Blocks	Length of Blocks	No. of Genes Affected	No. of SNPs on Coding Region	Example of Genes
34503-34793	290	1	11	
44406-49627	5221	3	220	*hemN_1, ligA_1, ZipA,*
56530-63726	7196	10	194	*kdsB, trpA, accD, tusA, pyrC, nusB*
446709-447979	1270	1	77	*PorA*
451130-452181	1051	1	32	*pepQ,*
461120-465066	3946	6	192	*dus_1, fis, ruvC, htrB_1*
754534-758680	4146	5	113	*fdx_1, hscA,*
1290053-1292695	2642	4	112	*ilvE, fabl*
1316226-1317578	1352	1	11	*vapA*
1361946-1367876	5930	4	235	*ostA, dsbA, mviN*
1456134-1461230	5096	6	105	*rnhB, soj, gidB, yeeA, frr*
1560953-1569496	8543	9	149	*galE_1, haeIIIM_2, tex, ctr operon*
1579243-1583633	4390	5	142	*rfbA, rmlC, dnaJ_2*
1856950-1857837	887	1	67	
1858952-1864826	5874	3	99	*pabB, ssa1*
1955526-1959383	3857	4	161	*yeaZ, fHBP, fda, xerC,*
2131346-2135326	3980	3	35	*ubiB, cysE, grpE*

Supplemental Table 5. Genetic changes identified from comparative WGS analysis of reference strain M5178 and University B outbreak isolate PMB4478. The list is restricted to SNP/indel events that alter protein sequences or are within 100 nucleotides of protein coding regions and have the potential to alter gene expression.

Gene ID	*Gene Name*	Genetic Changes	Gene Description
00003	*pglE*	promoter, T->C	Periplasmic protein
00056	*pilX*	S100P	minor pilin PilX,Pilin,putative major pilin subunit,Tfp pilus assembly protein
00079	-	K35E	putative phage repressor protein,Peptidase S24-like
00088	*icd*	promoter, T->C	isocitrate dehydrogenase
00128	*uvrA*	S849A	excinuclease ABC subunit A
00142	-	16, ins. A	Membrane protein,MAPEG family
00156	-	promoter, del. T	hemagglutinin family protein,Type V secretory pathway
00158	-	promoter, C->G, G->C	acyl-CoA dehydrogenase,Acyl-CoA dehydrogenase
00164	*dus_1*	R139S	tRNA-dihydrouridine synthase C
00191	*purK*	ins. A; ins. T	phosphoribosylaminoimidazole carboxylase ATPase subunit
00203	*leuB*	ins. G; ins. G	3-isopropylmalate dehydrogenase
00217	*copA_1*	del. A; ins. A	P-type cation-transporting ATPase,Probable copper-importing P-type ATPase A
00218	-	10 ins. C	hypothetical protein
00276	-	G150S	protein gp32,Mu-like prophage I protein
00285	-	Q99P	putative lipoprotein
00371	*hupB*	promoter, ins. A	DNA-binding protein Hu,DNA-binding protein HU-beta,transcriptional regulator HU subunit beta
00391	*narX_1*	357 ins. TCAAA	putative two-component system sensor protein
00475	-	K80Q	putative metallopeptidase
00498	-	38 del. A	hypothetical membrane protein
00516	-	169 ins. AGCCAGCCAGCC	restriction modification protein Mod,Probable hemoglobin and hemoglobin-haptoglobin-binding protein 3 precursor
00519	-	172 ins. T	type III restriction/modification system enzyme
00529	-	139 ins. A	transposase
00530	-	R119C	putative phase associated protein
00547	-	104 ins. G	hypothetical protein
00552	*frpC1*	I1401T; 1653 ins. A	putative RTX-family exoprotein,Cyclolysin,3-phytase (myo-inositol-hexaphosphate 3-phosphohydrolase)
00566	*porA*	A45S; promoter del. C	outer membrane porin protein
00575	*purE*	S114A	phosphoribosylaminoimidazole carboxylase catalytic subunit
00607	-	K67Q	hypothetical protein
00657	*icsA*	promoter del. A, del. TGCT	virulence associated protein,Outer membrane protein IcsA autotransporter precursor
00717	*hgd*	220 ins. A	3-hydroxyacid dehydrogenase,2-(hydroxymethyl)glutarate dehydrogenase
00745	*regA*	6 ins. CCCGTC	two component response regulator,Response regulator prrA,acetoacetate metabolism regulatory protein AtoC
00747	-	22 del. A	protein of unknown function
00810	*hpuB*	385 ins. C	hemoglobin-haptoglobin utilization protein B
00918	-	71 del. C	putative protein export protein
00919	-	N69K	putative protein export protein
00924	-	promoter del. T	cupin family protein
00933	*citN*	I68N	transmembrane transport protein,Citrate transporter,H /gluconate symporter and related permeases
00948	-	K72Q	PilP protein,Pilus assembly protein
00957	*pglC*	206 ins. A	pilin glycosylation protein,L-glutamine:2-deoxy-scyllo-inosose aminotransferase
00958	*capD*	N270D	UDP-N-acetylglucosamine 4,6-dehydratase,Polysaccharide biosynthesis protein
00960	-	H274Q	putative metal-dependent phosphoesterase,hypothetical protein
00973	-	promoter, del. C	putative lipo-oligosaccharide acyltransferase
01001	*miaB*	8 ins. A; A28S	dimethylallyl)adenosine tRNA methylthiotransferase
01041	-	V150I	hypothetical protein
01046	-	H11P	hypothetical protein
01060	*lgtA*	promoter, del. CCC	lacto-N-neotetraose biosynthesis glycosyl transferase
01062	-	70 del. C	Uncharacterized protein conserved in bacteria,Protein with unknown function
01100	*ssa1*	del. CCC; G226D	extracellular serine protease precursor,Serotype-specific antigen 1 precursor
01127	*yadA*	promoter, G->A	adhesin/invasin,Adhesin yadA precursor
01140	*hrpA`*	372 ins. A	ATP-dependent DNA helicase,ATP-dependent RNA helicase
01195	*petB*	350 ins. T	cytochrome B,Cytochrome b,cytochrome b6
01219	*birA*	109, ins. T	bifunctional biotin--[acetyl-CoA-carboxylase] ligase/pantothenate kinase
01264	-	D64G	hypothetical protein
01275	-	92 ins. G	hypothetical protein
01287	*dtpT*	M138V	Di-/tripeptide transporter,putative tripeptide transporter permease
01296	-	R93L	lipoprotein
01333	*pilE_7*	del. GGCGGT	fimbrial protein P9-2 precursor,Pilin,Tfp pilus assembly protein
01340	*glmS*	promoter, A->C	glucosamine--fructose-6-phosphate aminotransferase
01386	-	del. A; del. A	hypothetical protein

Gene ID	*Gene Name*	Genetic Changes	Gene Description
01386	-	del. A; del. A	hypothetical protein
01401	-	del. T; del. T	hypothetical protein
01434	*tufA_1*	ins. A	elongation factor Tu,GTPases - translation elongation factors
01449	*tufA_2*	ins. A	elongation factor Tu,GTPases - translation elongation factors
01464	*rplE*	V17F	50S ribosomal protein L5
01493	*rseP*	261 ins. T	integral membrane protein,Regulator of sigma E protease,zinc metallopeptidase
01530	-	245 ins. C	pilin glycosyl transferase A
01672	-	60 del. TT	hypothetical protein
01720	*mraY*	promoter, del. A	phospho-N-acetylmuramoyl-pentapeptide- transferase
01721	*hcpC*	107 ins. A	Sel1 repeat family protein,Putative beta-lactamase
01762	*potD-1*	34 ins. A	spermidine/putrescine ABC transporter substrate-binding protein
01804	-	130 ins. C	hypothetical protein
01830	*mucD*	K32Q	serine protease,Probable periplasmic serine endoprotease DegP-like precursor
01841	*lutP*	K386Q	L-lactate permease
01854	-	S79N	putative secreted protein
01880	-	3 ins. C	dissimilatory nitrous oxide reduction protein, lipoprotein,NosL
01893	-	S501A	putative N-acetyltransferase, acetyl coenzyme A synthetase
01959	-	N72K	mafB silent cassette
01999	*ksgA*	I35V	dimethyladenosine transferase,Ribosomal RNA small subunit methyltransferase A
02002	*iga_2*	704 ins. TTGCAGCTGAGTGG[illegible]	IgA-specific serine endopeptidase
02003	*iga_3*	K305{STOP]	IgA-specific serine endopeptidase
02058	*pbpG*	S101F	D-alanyl-D-alanine carboxypeptidase
02064	*purC*	promoter, ins. GG	phosphoribosylaminoimidazole-succinocarboxamide synthase
02101	-	ins. AAAGAGTGCG	VanZ family protein,Predicted integral membrane protein
02122	*purA*	promoter, T->C	adenylosuccinate synthetase
02139	*hsdR_2*	H164Y	putative type I restriction enzyme EcoR124II R protein
02140	*clpA*	147 del. CTTCAT	ATP-dependent protease ATP-binding protein

Supplemental Table 6. Recombination events identified from comparative WGS analysis of reference strain M5178 and University B outbreak isolate PMB4478. Only recombination blocks >100bp are listed.

Recombination Blocks	Length of Blocks	No. of Genes Affected	No. of SNPs on Coding Region	Example of Genes
17284-17771	487	2	3	
268417-269222	805	1	8	
272834-272989	155	1	9	
401844-402076	232	1	6	*mfd*
526156-527309	1153	1	39	*ilvD_2*
599406-601330	1924	2	44	*fpr_2, polC*
667627-672334	4707	5	66	*ackA_1, dipZ, slyD*
695283-700109	4826	2	257	*lbpA, tbpB_1*
794820-795071	251	2	12	
893495-894269	25034	1	8	*exbB, mexB*
895351-918529	32091	17	304	*mexA, acrR, abgT, recC, ccoP2, relA,czcD*
1046315-1047475	1160	1	143	*pilC*
1320069-1327524	7455	3	51	*tsf, pyrH, mafA1_1*
1627497-1637640	10143	12	374	*nuo operon, fic, ispA, xseB*
1644428-1658147	13719	11	293	*bioH, recQ, trpC, mviN, dsbA_1, ostA, prsA*
1691958-1692625	667	1	1	
1791886-1794817	2931	2	282	*penA, murE,*
1847754-1850989	3235	2	198	*tbpB_2, tbpA*
1942317-1943249	932	2	58	*adhA, pilV1,*
2197482-2199914	2432	4	60	*ftsH, rlmE, hemB*

Supplemental Table 7. Functional distribution of proteins altered by SNP, indel, or recombination events identified when comparing a representative university outbreak strain and the respective non-outbreak reference isolate.

Outbreak Strain	Reference Isolate	Number of Differences Identified in Each Functional Group (% of total)				
		Surface – Capsule - Virulence factor	Metabolism - Pathway	Transcription – Translation - Signalling	Transport - Other	Total
PMB5026	PMB5266	11 (9.3%)	34 (28.8%)	47 (39.8%)	26 (22.0%)	118
PMB4478	M5178	21 (12.1%)	38 (22.0%)	59 (34.1%)	55 (31.8%)	173

Supplemental Table 8. Sequence variation between Illumina MiSeq and PacBio WGS platforms

Sample Name	Number of PE reads mapped	Percentage of mapped reads	Fraction of reference covered (>10X)	Average coverage	Number of variations	Note
PMB5026	3,431,964	99.1%	94.6%	293	1	95100^95101, 1bp insertion (C)
PMB4478	3,053,156	97.8%	96.0%	271	1	1000008^1000009, 3bp insertion (AGC)
PMB4479	2,212,047	98.9%	96.1%	192	1	1000004^1000005, 2bp insertion (GG)
